# Technical Note of a 3D Printed Stimulating Palatal Plate Prototype for Children with Trisomy 21 

**DOI:** 10.4317/jced.62013

**Published:** 2025-02-01

**Authors:** Natasha Ferreira Roltenver do Nascimento, Flávio Warol, Pedro Lima, Lais David Amaral, Daiana Barrozo dos Reis, Bruna Lavinas Sayed Picciani

**Affiliations:** 1 DDS, MSc. Undergraduate Program in Dentistry, Nova Friburgo Health Institute, Fluminense Federal University, Nova Friburgo/RJ, Brazil; 2 DDS, MSc, PHD. Graduate School in Dentistry, Nova Friburgo Health Institute, Fluminense Federal University, Nova Friburgo/RJ, Brazil; 3 Graduate School in Dentistry, Nova Friburgo Health Institute, Fluminense Federal University, Nova Friburgo/RJ, Brazil; 4 DDS, MSc, PHD. Graduate Program in Dentistry, Catholic University of Brasilia, Brasilia, DF, Brazil; 5 DDS, MSc, PHD. Undergraduate School in Dentistry, Nova Friburgo Health Institute, Fluminense Federal University, Nova Friburgo/RJ, Brazil

## Abstract

**Background:**

The stimulating palatal plate is an orthopedic device indicated for babies with Trisomy 21. A digital workflow should be implemented to enhance infant safety, starting by intraoral scanning and ending with device printing using a biocompatible material. Thus, the main objective of this technical note is to describe the digital workflow for fabrication of a palatal plate for therapy in T21 patients.

**Material and Methods:**

The fully digital linear workflow is presented below, as well as the effectiveness of use in a clinical case of an infant with T21. The maxilla was registered using the TRIOS3 intraoral scanner and the design process for the Stimulating Palatal Plate (SPP) aligns with similar parameters to craft an individual tray, using the Exocad software. After completion of the printing phase, the devices go through a crucial post-processing stage.

**Results:**

Upon placement, the SPP was perfectly adapted and the child with T21 presented immediate alteration of the tongue posture and lip sealing.

**Conclusions:**

The digital workflow from scanning to impression of the stimulating palatal plate is efficient and safe and should replace the conventional method, especially in patients with Trisomy 21. The printed plate presents excellent adaptation and acceptance by family members.

** Key words:**Trisomy 21, Down Syndrome, Stimulating Palatal Plate, Digital workflow, Computer-aided impression (CAI), Castillo Morales, intraoral scanning.

## Introduction

In Trisomy 21 (T21), as in other syndromes, it is essential to initiate functional orthopedic treatment of the jaws since birth, due to factors that cause oromotor problems, observed during swallowing, chewing, speaking, and breathing (1,2). In these patients, conventional impression techniques may pose a risk to the infant health due to the high risk of material aspiration, thus it is safer and more reliable to use intraoral scanning (IOS) to obtain digital models (3). Also, the use of CAD-CAM technology has enabled new three-dimensional technologies for the treatment of children with T21 (4). The stimulating palatal plate is an orthopedic device indicated for infants with T21 introduced by Castillo-Morales that improves the function of the orbicularis oris muscle and mimics muscles, improving sucking, articulation, swallowing, and nasal breathing (5,6). To begin manufacturing this device, it is essential to implement a digital workflow to ensure the infant safety, starting by intraoral scanning and ending with device printing using a biocompatible material with mechanical resistance (7,8).

The main objective of this technical note is to describe how to implement digital workflow for fabrication of a palatal plate for therapy in T21 patients. Also, a fully digital linear workflow is presented, as well as the effectiveness of use in a clinical case of an infant with T21.

## Material and Methods

-Intraoral scan

**Figure 1:**
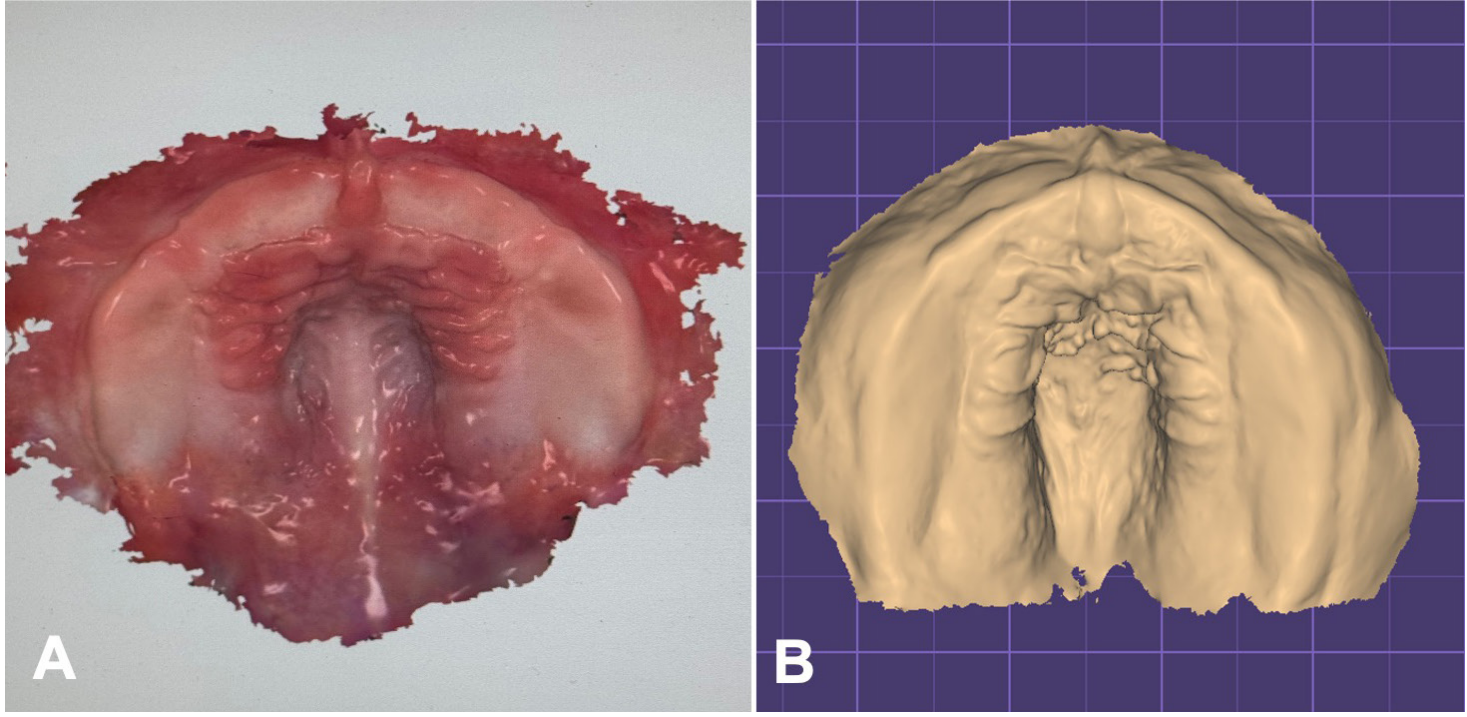
A. Maxillary intraoral scan and B. Finished digital model.

The maxilla was registered using the TRIOS3 intraoral scanner (3Shape, Copenhagen, Denmark) and the TRIOS Scanning Software. The scanning time was 1 min, registering 258 pictures. During the scan, the professional was careful to include all areas that could influence the fit of the plate. The most important structures included the maxillary tuberosity, the vestibule and the labial frenulum. The scanner needs a defined reference point at which the scan is always started, which was taken at the incisive papilla. Before model creation, the raw scan was automatically refined by applying the “postprocessing tool” (Fig. 1A).

-Design of a working model

After importing the scan data into the 3Shape Ortho Appliance designer, the digital model was exported as a Standard Tessellation Language (STL) file to the Exocad program (Fig. 1B).

-Design of a Stimulating Palatal Plate

**Figure 2:**
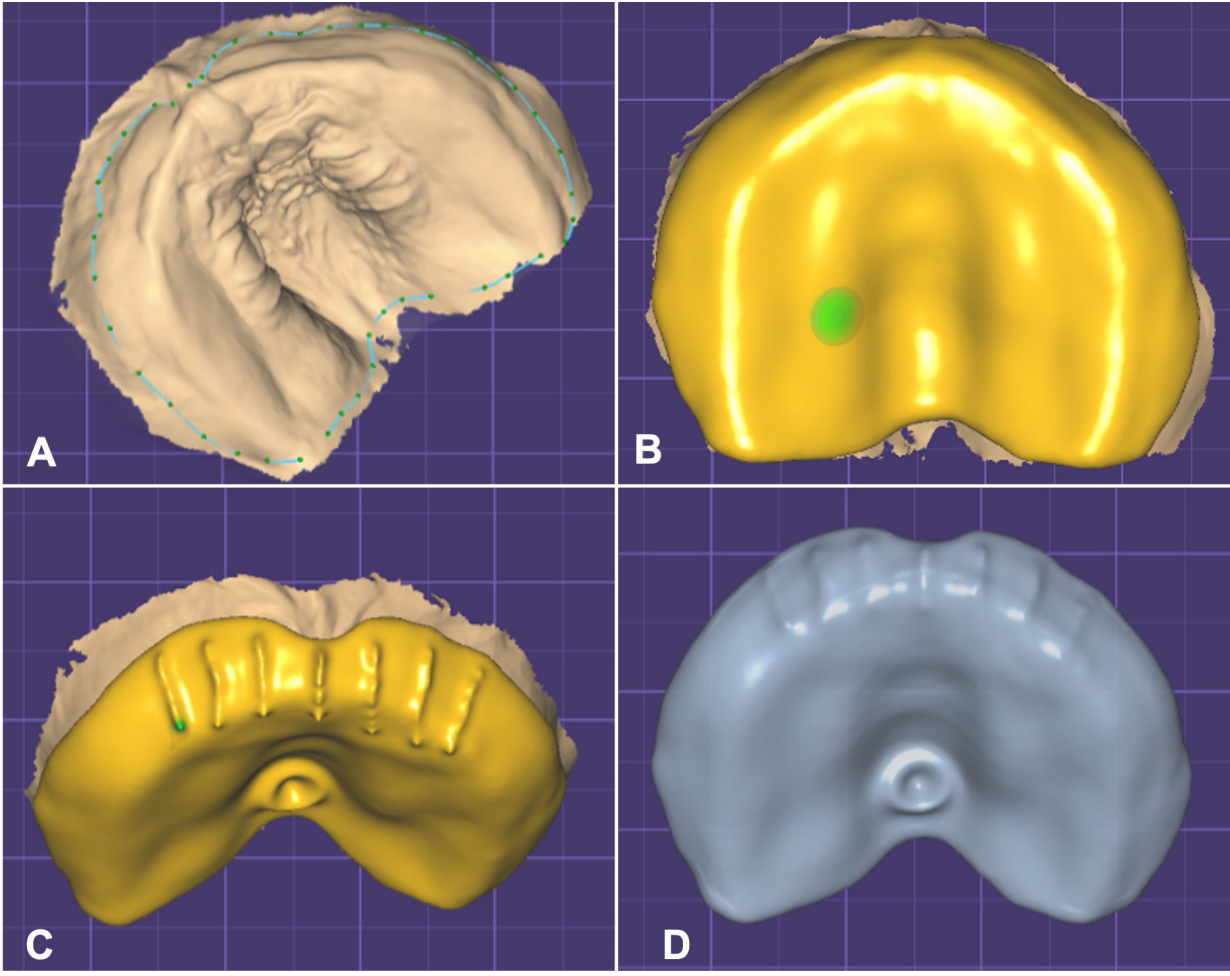
SPP design phase on the prepared model file: A. Contour of the Stimulating Palatal Plate. B. Completing the drawing. C. and D. Delineating the buccal grooves and palatal button.

After receiving the STL file derived from intraoral scanning using the TRIOS3 intraoral scanner, the design process for the Stimulating Palatal Plate (SPP) aligns with similar parameters to craft an individual tray or interocclusal device for bruxism treatment, utilizing the Exocad software. The initial step involves registering essential details such as the patient, operator, and the nature of the intended work. Initiating by model preparation, adjustments are made to the base by refining parameters and eliminating excesses from the scanning process. This phase involves fine-tuning the model in terms of thickness, base size, and identification. Subsequently, the operator progresses to the SPP design phase on the prepared model file, featuring a streamlined base without excesses, enabling a comprehensive visualization of all necessary margins crucial for fabrication of the SPP (Fig. 2A). Drawing parameters afford flexibility for modifications and adjustments, thereby facilitating an optimal result. Before onset, the operator has the option to fine-tune occlusal and peripheral thickness, as well as the minimum thickness of the SPP. Upon completing the drawing, the subsequent phase involves delineating the palatal button and buccal grooves, achieved by judicious addition and subtraction of volume from the SPP (Fig. 2B). The palatal button has a diameter of 5mm and height of 3mm, and the plate thickness is 2mm. Seven buccal grooves are made, without vertical tips, 2mm thick and wide, using the midline as reference to make the first and three more on each side at a 4-mm distance between them (Fig. 2C). After concluding this stage, the finalized SPP is stored within the software, rendering the file ready for the printing process (Fig. 2D).

-Stimulating Palatal Plate Printing 

**Figure 3:**
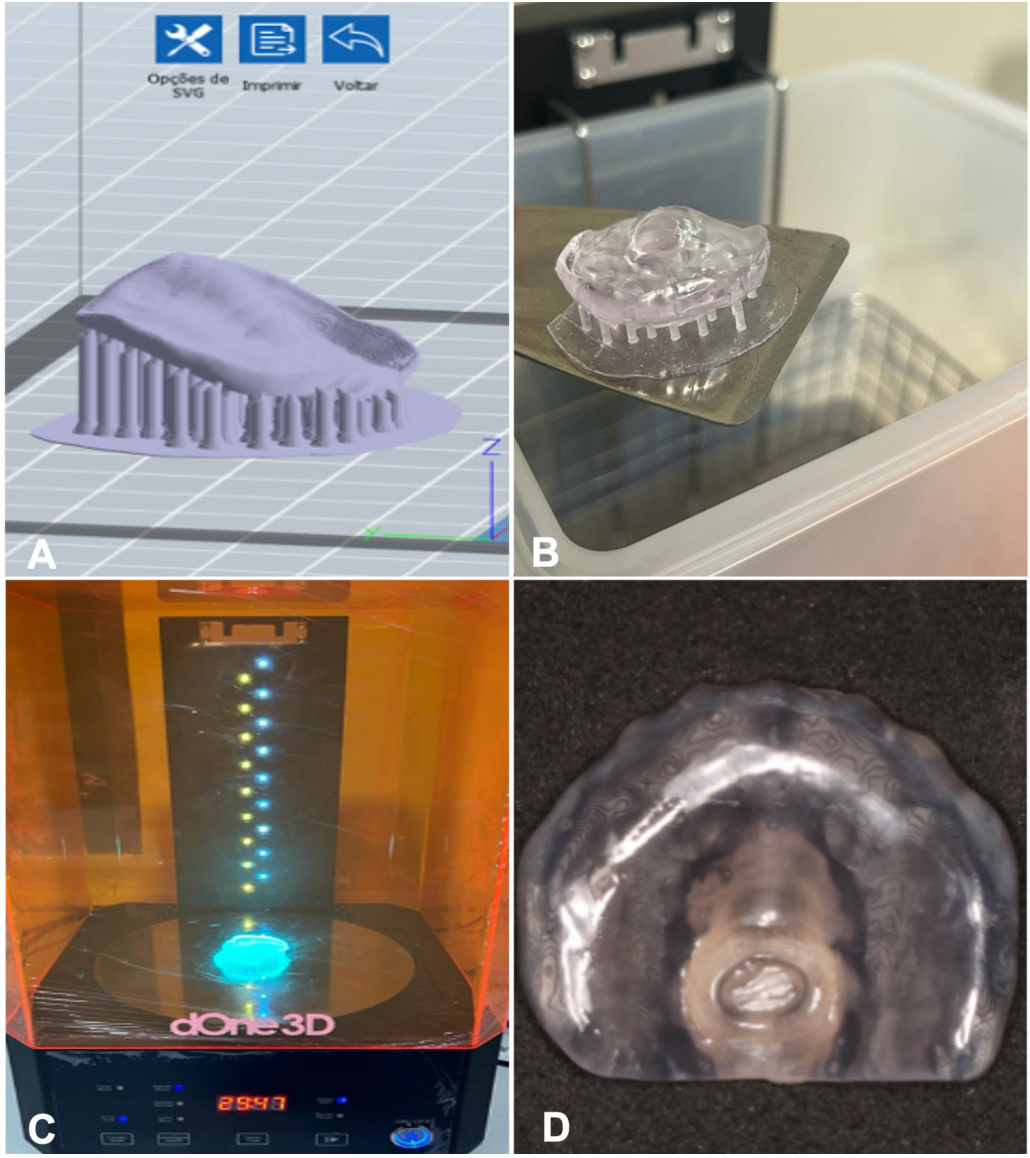
Stimulating Palatal Plate Printing and Post-Processing: A. Positioning the model on the printing platform and creating supports to anchor the initial layers of the base model. B. Plate printing before post-processing. C. Plate into a UV light chamber for post-curing. D. The finalized SPP is printing.

To generate the SPP, specialized software designed for 3D printing is employed, distinct from general design software. Typically tailored for 3D printers, this software facilitates the three-dimensional positioning of models, simulating the print space specific to each printer model. Each printer, in turn, accommodates a defined number of models for printing. The adjustment parameters are customized based on the resin chosen for each model, and these settings are stored in the software for ease of use in subsequent prints (Fig. 3A). After transferring the .stl file from the design program to the printer software, the following steps involve positioning the model on the printing platform and creating supports to anchor the initial layers of the base model. A crucial outcome of this process is the presentation of vital information on the time and quantity of resin to be utilized. This is instrumental in calculating the overall cost of the printing task. After completion of this stage, the file is ready and can be seamlessly sent to the printer to initiate the printing process (Fig. 3B).

-Stimulating Palatal Plate Post-Processing

After completion of the printing phase, the devices go through a crucial post-processing stage. This phase involves washing the parts using specific solutions determined by the resin manufacturer, such as water and alcohol in various concentrations. An essential tool in this process is the washing/curing machine, whose function is to immerse the piece in a cleaning solution and then perform a polymerization cycle to conclude the process. After printing, the objects are placed in an automatic washing machine immersed in isopropyl alcohol for 5 minutes in motion to remove the resin from the surface and dry completely. Then, the objects are inserted into a UV light chamber for post-curing for 20 minutes (Fig. 3C) and then submitted to mechanical polishing, following the manufacturer’s instructions of Prizma 3D Biosplint (Makertech Labs). This is vital because the layer-by-layer polymerization performed during printing is not sufficient to completely finalize the device. After concluding this stage, the finalized SPP is printing (Fig. 3D).

Results

**Figure 4:**
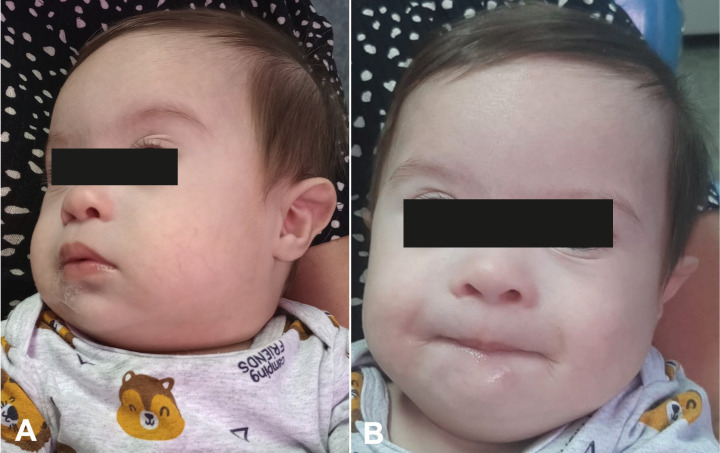
A. Stimulating Palatal Plate perfectly adapted. B. The child presented lip sealing.

This paper was approved by the Institutional Review Board (CAAE 60151022.0.0000.5626 – evaluation report: 5.603.099). The participant’s caretaker signed an informed consent form and an authorization form for the use of images and voice sound for research purposes, agreeing to the report and publication of results. A 4-month-old male participant diagnosed with T21 attended the Functional Jaw Orthopedics Clinic for Patients with Special Needs at Fluminense Federal University, Rio de Janeiro, Brazil. During the initial consultation, the child was evaluated by a specialist in patients with special needs and an orthodontist. Extraoral examination revealed hypotonia of the lips and tongue, absence of lip sealing, protruding and immobile tongue, drooling and everted lower lip. Intraoral examination evidenced a high-arched palate with maxillary atresia. Photographic and video recordings of the participant’s face were achieved to document the usual lip and tongue position before treatment. For video recording, the child was asked to sit on the caretaker’s lap, without any type of interaction. After evaluation, the use of printed SPP was indicated to treat lip and tongue hypotonia. The maxilla was scanned using a TRIOS3 intraoral scanner (3Shape, Copenhagen, Denmark) and the TRIOS Scanning Software. The STL file was sent to the laboratory for model printing and SPP fabrication. A printed SPP with 2 mm thickness was used, including seven buccal grooves with 2 mm height and width, the first using the midline as reference and three more in posterior direction on each side at a 4-mm distance between them to stimulate the upper lip. The plate also featured a palatal button with 3-mm height and 5-mm width, located at the limit of the soft palate. At the consultation for SPP placement, the caretaker received the following instructions: the child should only use the SPP under adult supervision, for a maximum daily period of 2 hours, divided into 4 sessions of 30 minutes. Upon placement the SPP was perfectly adapted, and the child presented immediate changes in tongue posture and lip sealing (Fig. 4A,B). Chairside adaptation of the plate was not necessary. The caretaker was instructed to use an adhesive if the SPP became loose during muscle movements of the tongue and lip, and to return for monthly follow-up. After the first month, the participant returned for follow-up and the caretaker reported use for the indicated period and observation of improvement in lip and tongue hypotonia.

## Discussion

There is lack of studies in the literature reporting the digital workflow in the routine care of infants and children with Trisomy 21, and it is essential to demonstrate how the digital workflow can positively impact this care. The present case reports the fabrication of a stimulating palatal plate in a fully digital workflow, found only in two studies by the same group (4,9).

Oral scanning in infants is a quick and safe procedure, without risk of material aspiration and model distortion. Also, in non-cooperative patients, despite all difficulties, it is still more comforTable and efficient than traditional impression. Traditional molding may be unsatisfactory, requiring repetition, which causes discomfort for babies and distress for parents (4,10). In this case, it was difficult to control the saliva and it was necessary to use a mouth opener to maintain the mouth opening, thus achieving faster scanning with more reliable image generation. Studies report that professionals performing scanning on infants prefer scanners with smaller heads (10-13). Additionally, the more experienced the professional performing the scanning, the less difficult and time-consuming the process (14). The area to be scanned for perfect plate adaptation is essential, and excellent communication between clinician and technician is necessary. The buccal folds determine the maximum plate height, and the limit between hard and soft palate determine its length (4). The scanner model can also influence the plate quality, reducing the need for adjustments to the plate, as in the present case. In a comparison of different intraoral scanners, the TRIOS 3 performed better in scanning speed and accuracy (15).

Due to the accuracy of the design and printing plate process, adjustments to the printed plate are often unnecessary. In this case, the plate adapted perfectly, and the patient was able to use it without adhesives. The post-processing stage of the plate influences the plate stability and durability, and it is essential to follow the manufacturer’s instructions of the chosen resin, both concerning washing time and light curing (4). Important aspects for plate retention include the thickness and smoothness; the greater the thickness, the greater the weight, impairing the plate retention. Also, the device must have an extremely smooth surface to avoid injuries to the baby; however, this reduces plate retention (4). Thus, a thinner device (2 millimeters) provides better results considering the physical factors of retention and stability.

A major disadvantage of the printed plate is the impossibility of inserting expanding screws to follow the maxillary growth and increase the plate durability (4). This patient must be followed, and new plates must be made. In this case, the patient is undergoing monthly follow-up, the plate is adapted, and the family is satisfied with the outcome. Some studies report the effectiveness of the stimulating palatal plate in older patients, over one year of age; at this stage, new plates or adjustments are necessary due to the intense period of tooth eruption (16,17).

## Conclusions

The digital workflow from scanning to printing the stimulating palatal plate is efficient and safe and should replace the conventional method, especially in patients with Trisomy 21. The printed plate presents excellent adaptation and acceptance by the family members. However, longitudinal follow-up studies should be performed to better understand the plate stability.

## Data Availability

The datasets used and/or analyzed during the current study are available from the corresponding author.
